# Effectiveness of information and communication technologies to improve the knowledge of hospitalized patients: systematic review

**DOI:** 10.15649/cuidarte.3854

**Published:** 2024-08-13

**Authors:** Maria Aline Moreira-Ximenes, Natália Ângela Oliveira-Fontenele, Maria Girlane Sousa-Albuquerque Brandão, Francisca Elizângela Teixeira-Lima, Samila Gomes Ribeiro, Cristiana Brasil de Almeida-Rebouças, Lívia Moreira-Barros, Joselany Áfio-Caetano

**Affiliations:** 1 Universidade Federal do Ceará, Fortaleza - CE, Brazil. E-mail: aline.ximenes11@hotmail.com Universidade Federal do Ceará Universidade Federal do Ceará Fortaleza Brazil aline.ximenes11@hotmail.com; 2 Universidade Federal do Ceará, Fortaleza - CE, Brazil. E-mail: nataliaaof@hotmail.com Universidade Federal do Ceará Universidade Federal do Ceará Fortaleza Brazil nataliaaof@hotmail.com; 3 Universidade de São Paulo, São Paulo, Brazil. E-amil: girlanealbuquerque@usp.br Universidade de São Paulo Universidade de São Paulo São Paulo Brazil girlanealbuquerque@usp.br; 4 Universidade Federal do Ceará, Fortaleza- CE, Brasil. E-mail: felisangela@yahoo.com.br Universidade Federal do Ceará Universidade Federal do Ceará Fortaleza Brazil felisangela@yahoo.com.br; 5 Universidade Federal do Ceará, Fortaleza - CE, Brazil. E-mail: samilagomesribeiro@gmail.com Universidade Federal do Ceará Universidade Federal do Ceará Fortaleza Brazil samilagomesribeiro@gmail.com; 6 Universidade Federal do Ceará, Fortaleza- CE, Brazil. E-mail: cristianareboucas@yahoo.com.br Universidade Federal do Ceará Universidade Federal do Ceará Fortaleza Brazil cristianareboucas@yahoo.com.br; 7 Universidade da Integração Internacional da Lusofonia Afro-Brasileira, Redenção - CE, Brazil. E-mail: livia@unilab.edu.br Universidade da Integração Internacional da Lusofonia Afro-Brasileira Universidade da Integração Internacional da Lusofonia Afro-Brasileira Redenção Brazil livia@unilab.edu.br; 8 Universidade Federal do Ceará, Fortaleza- CE, Brazil. E-mail: joselany@ufc.br Universidade Federal do Ceará Universidade Federal do Ceará Fortaleza Brazil joselany@ufc.br

**Keywords:** Health Education, Information and Communication zechnologies, Health Promotion, Nursing Care, Educación para la Salud, Tecnologías de la Información y la Comunicación, Promoción de la Salud, Cuidado de Enfermería, Educação em Saúde, Tecnologias de Informação e Comunicação, Promoção da Saúde, Cuidados de Enfermagem

## Abstract

**Introduction:**

Hospitals serve as important settings for health education initiatives facilitated by technology. Utilizing these resources, healthcare professionals can enhance patient care practices.

**Objective:**

This study aims to assess the efficacy of information and communication technologies in enhancing the knowledge of hospitalized patients.

**Materials and Methods:**

A systematic review was conducted to address the research question: "Which information and communication technologies effectively improve the knowledge of hospitalized patients?" The search encompassed electronic databases such as Scopus, the National Library of Medicine, Web of Science, Cumulative Index of Nursing and Allied Health Literature, Scientific Electronic Library Online, Cochrane, and Excerpta Medica Database. Gray literature was sourced through Google Scholar. The exposure factor was educational interventions employing information and communication technologies, with the outcome measured as improved knowledge. Only randomized clinical trials in any language were included.

**Results:**

A total of 1,153 articles were initially identified, from which four were deemed eligible for inclusion. These studies demonstrated effectiveness in enhancing knowledge through educational interventions utilizing technologies such as video, applications, and concept maps.

**Discussion:**

Information and communication technologies have been shown to enhance patient knowledge. Nonetheless,there is a paucityofresearch exploring hospitals as platforms for technology-mediated educational interventions.

**Conclusion:**

Educational interventions employing information and communication technologies in hospital settings effectively enhance patient knowledge.

## Resumen

**Introducción:**

El hospital es un escenario para el desarrollo de acciones de educación en salud mediadas por tecnologías. El uso de estos recursos por profesionales puede contribuir a mejores prácticas de salud.

**Objetivo:**

Evaluar la eficacia de tecnologías de información y comunicación en la mejora del conocimiento de pacientes hospitalizados.

**Materiales y Métodos:**

Revisión sistemática realizada a partir de la pregunta: ¿Qué tecnologías de información y comunicación son eficaces en la mejora del conocimiento de los pacientes hospitalizados? La búsqueda se realizó en las bases de datos Scopus, National Library of Medicine and National Institutes of Health, Web of Science, Cumulative Index of Nursing and Allied Health Literature, Scientific Electronic Library Online, Cochrane y Excerpta Medica Database. Para la búsqueda de literatura gris, se utilizó Google Scholar. El factor de exposición fue la intervención educativa con uso de tecnologías de información y comunicación, y como resultado: mejora del conocimiento. Se incluyeron solo ensayos clínicos aleatorizados, en todos idiomas.

**Resultados:**

Se identificaron 1 153 artículos, de los cuales se incluyeron cuatro que revelaron eficacia en la mejora del conocimiento a partir de intervenciones educativas mediadas por tecnología del tipo video, aplicación y mapa conceptual.

**Discusión:**

Las tecnologías de información y comunicación mejoran el conocimiento de los pacientes; sin embargo, son pocos los estudios que exploran el hospital como medio para realización de intervenciones educativas mediadas por tecnologías.

**Conclusión:**

Las intervenciones educativas con el uso de tecnologías de información y comunicación en el ámbito hospitalario son eficaces para la mejora del conocimiento de pacientes.

## Resumo

**Introdução:**

O hospital é um dos cenários para o desenvolvimento de ações de educação em saúde mediadas por tecnologias. O uso destes recursos pelos profissionais pode coadjuvar em melhores práticas de saúde.

**Objetivo:**

Avaliar a eficácia de tecnologias de informação e comunicação na melhora do conhecimento de pacientes hospitalizados.

**Materiais e Métodos:**

Revisão sistemática, realizada a partir da questão norteadora: quais tecnologias de informação e comunicação são eficazes na melhora do conhecimento de pacientes hospitalizados? A busca ocorreu nas bases de dados eletrônicas: Scopus; National Library of Medicine and National Institutes of Health; Web of Science; Cumulative Index of Nursing and Allied Health Literature; Scientific Electronic Library Online; Cochrane e Excerpta Medica dataBASE. Para a busca da literatura cinzenta, utilizou-se o Google Scholar. O fator de exposição foi a intervenção educativa com o uso das tecnologias de informação e comunicação, e como desfecho: melhora do conhecimento. Incluíram-se apenas ensaios clínicos randomizados, em todos os idiomas.

**Resultados:**

Identificaram-se 1.153 artigos, dos quais foram incluídos quatro que revelaram eficácia sobre melhora do conhecimento a partir de intervenções educativas mediadas por tecnologia do tipo vídeo, aplicativo e mapa conceitual.

**Discussão:**

Tecnologias de informação e comunicação melhora o conhecimento dos pacientes, no entanto, são poucos os estudos que exploram o hospital como meio para realização de intervenções educativas mediadas por tecnologias.

**Conclusão:**

As intervenções educativas com o uso das tecnologias de informação e comunicação no âmbito hospitalar são eficazes para a melhora do conhecimento dos pacientes.

## Introduction

In the realm of healthcare, hospitals have become settings for the development of actions utilizing various technologies for health education^1^^, ^^2^. Therefore, professionals’ sensitivity to the use of Information and Communication Technology (ICT) in healthcare can influence best health practices^3^.

A study conducted in the United Kingdom emphasizes that information and communication are significant components of interactions between patients and professionals in the emergency sector^4^. In Brazil, a study supports these findings by highlighting that ICTs are tools used by professionals for health education, providing convenience and greater accessibility to various topics addressed in this work process^5^.

Advancements in health information technology create opportunities for patients to actively engage in their care. Systematic reviews confirm these findings, showing that technology-based health interventions have had positive effects on patient engagement, behaviors, and knowledge^6^^, ^^8^.

Few studies have explored the use of ICTs to improve patient knowledge of health topics during hospitalization^7^, despite strong guidelines advocating for patient engagement^9^. Review studies that explored the use of ICTs in hospitals did not specifically address the effectiveness of these technologies aimed at improving patient knowledge^10^.

Furthermore, available studies touch upon ICT themes but seldom explore their relationship with knowledge and the types and aspects of interventions that yielded positive results. In this context, it becomes relevant to gather and synthesize evidence on the effectiveness of ICTs in improving the knowledge of hospitalized patients to support healthcare systems in Latin America, where challenges persist and resources are limited. Thus, the use of ICTs can assist in improving patient care and the culture of patient safety^11^^, ^^12^.

This review aims to provide healthcare professionals, researchers, and policymakers with an overview of interventions aimed at improving knowledge, their procedures, benefits, and application scenarios. Therefore, the objective is to evaluate the effectiveness of information and communication technologies in enhancing the knowledge of hospitalized patients.

## Materials and Methods

This systematic literature review was conducted following the Joanna Briggs Institute (JBI) guidelines and registered in the PROSPERO platform under registration CRD42023410493. The Preferred Reporting Items for Systematic Reviews and Meta-Analyses (PRISMA) was used to report this review^13^.

The research question was guided by the PICO framework. It was formulated as follows: Population (P) - hospitalized patients; Intervention (I) - information and communication technologies for health education; Comparator (C) - not defined as it varied across studies; and Outcome (O) - improvement in patient knowledge. Based on this framework, the following research question was developed^14^: Which ICTs are effective in improving the knowledge of hospitalized patients?

Studies in all languages were included if they assessed the effectiveness of ICTs used in hospitals to improve patient knowledge of health topics. Educational interventions using ICTs were required to begin and end in the hospital setting. The thematic focus of interventions was unrestricted, aiming to broaden the analysis on health education in hospitals, an area noted for existing knowledge gaps in the literature^10^^, ^^15^. The established outcome was an improvement in patient knowledge. Exclusion criteria encompassed articles on ICT use in other settings, editorials, and letters to the editor.

The search for studies was conducted in April 2023 across the following databases: Scopus; National Library of Medicine and National Institutes of Health (PubMed/PMC); Web of Science; Cumulative Index of Nursing and Allied Health Literature (CINAHL); Scientific Electronic Library Online (SciELO); Cochrane, and Excerpta Medica Database (Embase). Grey literature was searched using Google Scholar.

The keywords from the PICO framework were used to identify controlled vocabulary terms in Health Sciences Descriptors (DeCS), Medical Subject Headings (MeSH), and Emtree. Preliminary searches in databases aimed to identify free vocabulary also used in the literature.

The search strategy developed was tested and approved across all selected databases, described as follows: (("Hospital" OR "Patient Engagement" OR "Patient Involvement" OR "Patient Participation") AND (“Information Technology” OR “Health Information Technology” OR "educational technology" OR "instructional technology") AND (“Knowledge” OR "Education, Health" OR "Health Education" OR "health promotion" OR "patient education")).

Citations and abstracts found were exported to Rayyan QCRI from the Qatar Computing Research Institute^16^ for removal of duplicate publications. Subsequently, the process of screening titles and abstracts was conducted independently by two reviewers. In cases of doubt or disagreement regarding the inclusion of any material, a third reviewer was consulted. References of included studies were then checked to identify additional studies.

The JBI instrument for data collection and analysis was adapted for this study, with the following data collected: study details (article title, journal, country, language), author/year, study design, objectives, population, sample losses, study period, setting/context, intervention description, comparator, evaluation method (instruments used to measure knowledge), number of sessions/intervention duration, assessment points, findings/conclusions, and contributions to practice.

For the final analysis of articles, the JBI Data Extraction Form for Experimental/Observational Studies was utilized. At this stage, both evaluators independently conducted methodological critical appraisal, considering common conclusions between them.

To synthesize the included articles, the following steps were followed: synthesis of outcomes, extraction of quantitative data, summarization of effect measures, flowchart of article selection in a figure, and presentation of descriptive data in tables^14^. After analysis, articles were categorized with the following information: country, sample, hospital sector, ICTs, duration and intervention details, technology used in the control and intervention groups, and key results.

Subsequently, the studies underwent rigorous methodological quality analysis using the JBI critical appraisal checklist for randomized controlled trials, consisting of 13 questions related to internal validity and statistical aspects of the studies. It is noted that all collected data are fully accessible and available for consultation on Figshare^17^.

## Results

**Figure 1:**
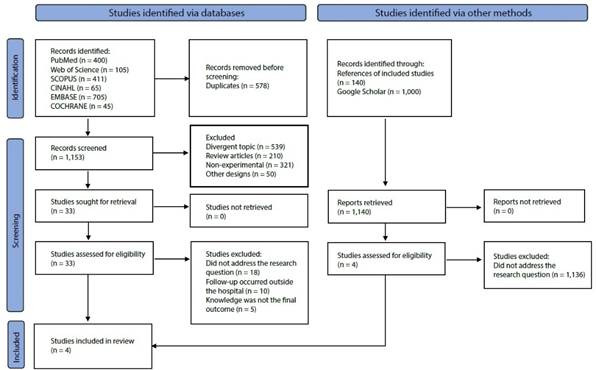
PRISMA flowchart of studies included in the review

From the search, 2,293 articles were identified, of which 578 were duplicates and 2,265 did not meet the eligibility criteria. Therefore, 37 studies were fully analyzed, and after this stage, a final sample of four studies was selected. The selection process can be observed in the flowchart (Figure 1).

The four articles selected for the final sample are from international journals published in English. The publications originated from Taiwan (1), Canada (1), Finland (1), and the United States (1). The four studies involved approximately 273 patients, predominantly adult females. The follow-up time for interventions ranged from one to 28 days. The following table (Table 1) presents details regarding the characterization of findings.

**Table 1:** Characterization of studies based on intervention information

Reference/ Country	Sample	Hospital sector	ICTs	Duration of intervention	Details of the educational intervention	Control	Instrument for measuring knowledge	Main results
Chang et al.,	70	Plastic surgery	Mobile	An average of	The intervention was	Brochure	Wound Care	Knowledge improved in both
2019 (Taiwan)^18^	participants	ward	application	28 days, with intervention at baseline, an average of nine days after and seven days before discharge	carried out by the nurse and mediated by ICT, with the aim of guiding the patient through dressing changes. During the procedure, photographic records of the step-by-step were made using the app, so that the patient could reinforce their knowledge along with the general instructions also present in the technology.		Knowledge Scale and Wound Care Skills Scale	groups, but the magnitude of the findings was greater in the intervention group (CI = 1.51, p = 0.025). Consequently, dressing change skills were also better in the intervention group (CI = 2.03, p = 0.001).
Mednick et	78	Department of	Educational	It took	The ICT aimed to guide	Standard	Knowledge test	The intervention group scored
al., 2016 (Canada)^19^	participants	phthalmology	video	place in one session.	patients through the Fluorescent Retinography (FR) procedure. The intervention was applied at the time of signing the consent form for the examination. The video was about four minutes long and presented information on clinical indications, risks, benefits, and how to administer (FR).	guidance offered by the ophthalmologist before the procedure	designed by the authors with six questions and four answer options.	better in the knowledge test (84.00% correct) than the control group (73.00% correct). Therefore, there was evidence of efficacy in improving knowledge with the use of video compared to standard guidance (p=0.001).
Johansson et al.,2007 (Finland)^20^	123 participants	Surgical clinic	Concept map	It took place in one session, lasting one hour, two weeks before admission for orthopedic surgery.	The intervention was applied by a nurse who had previously been trained to build a concept map with the aim of guiding patients through orthopedic surgery. The facilitator sat next to the patient and discussed functional, experiential, ethical, social, and financial issues associated with the surgery. As the intervention unfolded, the concept map was constructed and then made available to the patient.	Routine unit orientation	Orthopedic Patient Knowledge Questionnaire (OPKQ) Modified Empowerment Questionnaire (MEQ)	Patients who received pre admission health education had higher knowledge scores in both assessments, at admission and discharge (M = 4.05 > 4.30, improving 0.25, p < 0.002).
Rossi et	150	Health	Educational	It took	The intervention was	Routine unit	Knowledge	Patients in the intervention
al.,2005 (United States)^21^	participants	education sector in the hospital	video	place in one session	mediated using an educational video aimed at guiding patients through knee arthroscopy. The ICT was developed by the American Academy of Orthopedic Surgeons and lasted 12 minutes. After viewing the video, the patient asked questions of the intervention facilitator.	orientation	assessment questionnaire constructed by the authors	group obtained an average knowledge score of 81.00% in the knowledge test (SD=10.50%), while patients in the control group scored 71.10% (SD=14.10%). It was therefore observed that the video was effective in improving knowledge about the surgery (p=0.002)

*ICT: Information and communication technology.*

Regarding the study population, only one study^19^ had a majority of elderly individuals over 65 years old. The others were conducted with an adult population between 18 and 57 years old. Educational attainment was reported in three studies^18^^, ^^20^^, ^^21^, with a predominance of individuals having up to 12 years of schooling. No statistical associations were established between educational attainment and improvements in knowledge.

No cognitive assessment instruments were used to evaluate patients included in the studies. Additionally, sample losses were not significant, which may be attributed to the timing of the interventions, predominantly occurring in a single session with evaluations over a short period of time (up to 28 days).

**Figure 2:**
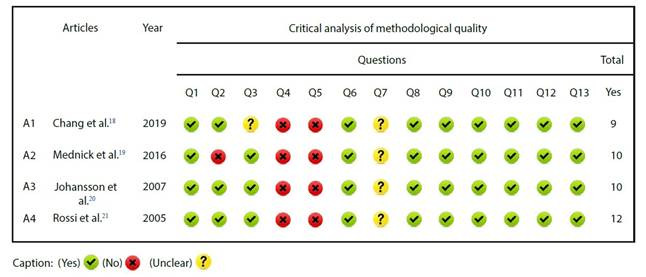
Quantitative evaluation of methodological quality in studies according to the JBI critical appraisal checklist

Regarding educational interventions, most took the form of individual counseling and discussions with patients. Printed resources such as concept maps^19^, audiovisual materials like videos^19^^, ^^21^, and digital technologies^18^ were employed. Among the topics covered in the educational interventions were instructions aimed at improving knowledge about wound care for home follow-up and aspects related to surgical procedures, fluorescent retinography, orthopedic surgery, and knee arthroscopy, which participants were to undergo. Regarding the professional who conducted the intervention, two studies^18^^, ^^20^ reported the involvement of nursing professionals, while in the others, the category was not specified. The rigorous analysis of methodological quality of the studies can be observed in Figure 2.

The method for sequence generation and allocation concealment of patients was not clearly described in the findings of some studies, nor was the blinding of outcome assessment for defined outcomes. This is attributed to the educational intervention being conducted at the bedside. Additionally, some studies exhibited a potentially high risk of bias due to insufficient description of the knowledge assessment methods.

## Discussion

Findings demonstrated that the use of ICTs to enhance knowledge in hospitals is highly promising. However, few studies explore these spaces as a means for conducting educational interventions mediated by technology. Conversely, hospitals still serve as places where participants are enrolled for follow-up interventions that take place in other settings.

Within the hospital setting, this idealization is reaffirmed by the Budapest Declaration, emphasizing the relevance of active patient profiles in tertiary care sectors and the improvement in the proposal and quality of health information, communication, and education programs^10^. Despite the existence of international guidelines^9^^, ^^22^, few studies have explored hospitalized patient knowledge as a strategy to enhance participation and self-care during hospitalization.

In the studies included in this review, the use of digital technologies such as mobile applications and videos stood out. These resources proved effective in enhancing patient knowledge. Digital tools are generally well-received due to their ease of use, integration of visual and auditory resources, and other features that enhance accessibility^23^. Other studies using mobile applications for health interventions highlight positive impacts and efficacy in health contexts^24^^, ^^26^.

The use of educational interventions through smartphones aligns with the increasing number of individuals seeking health information via mobile devices. It is believed that the functionalities of these apps possess important attributes for transforming healthcare practices, resulting in improved individual knowledge^10^^, ^^27^.

A noteworthy point was the use of theoretical frameworks for the development and application of ICTs. In this study, the self-regulation theory, which is based on self-monitoring, self-judgment, and self-reaction^28^ was applied. The use of this framework facilitated goal setting and strategy definition within the app to promote wound care self-management. However, only this study utilized a theoretical framework to underpin the educational intervention.

In studies using video to guide patients through surgical procedures, the influence ofthis resource on knowledge retention was evaluated compared to routine service guidance. Findings indicated that the intervention group achieved better knowledge retention among participants^19^^, ^^21^.

Another highlighted finding was patient satisfaction with ICT use. A study included in the review evaluated this outcome and demonstrated satisfaction among most participants in the intervention group^19^. These findings align with other systematic reviews on health technologies based on educational videos, which show their significant role in education, self-management, satisfaction, and improvements in patients' quality of life^29^^, ^^30^.

Overall, studies underscore that the use of videos and mobile applications improves patient knowledge, satisfaction, and accessibility, promotes active participation, and facilitates more effective communication during hospitalization^31^^, ^^32^. In addition to digital technologies, one study in this review applied a printed technology known as a concept map. This visual tool illustrates relationships between ideas and concepts, aiding in organizing and structuring knowledge and showing interconnections among different pieces of information^33^.

Research evaluating the use of concept maps for patient health education found that they significantly contributed to surgical patients' understanding of procedures^22^. This aligns with studies demonstrating that concept maps are widely used for teaching and learning, encouraging active participation and providing a comprehensive view of information^34^^, ^^35^.

It is crucial to emphasize the role of healthcare professionals as mediators of intervention. In all studies in this review, a professional was involved in the ICT application process, with nurses predominantly featured^18^^, ^^19^^, ^^21^. These findings support the nurse's role as an educator and underscore the importance of a multidisciplinary team in the educational process^7^.

Another relevant point was the measurement of patient knowledge and the evaluation of instruments. In two studies in this review, the authors developed their own instruments without reporting their validation, potentially compromising the internal validity of the findings^19^^, ^^21^. Using validated instruments ensures that the information obtained is reliable and contributes to better clinical outcomes^35^.

The review's limitations relate to the heterogeneity of the studies, which precluded meta-analysis execution, emphasizing the need to interpret findings with caution. Although educational interventions were heterogeneous, differences among the included studies should be noted. Some studies defined a small sample size and demonstrated less rigorous data analysis, challenging result validity.

Furthermore, this review provides valuable insights into intervention strategies that have proven effective in the hospital setting. Thus, its results can guide future interventions using technology to enhance patient knowledge about their care during hospitalization.

## Conclusion

Educational interventions using ICTs in the hospital setting effectively improve the knowledge of hospitalized patients, resulting in patient satisfaction, skill acquisition, and reduced anxiety among patients.

However, despite the numerous benefits of ICTs, it is important to emphasize that ICT implementation during hospitalization requires behavioral changes from both professionals and patients towards health education. Moreover, it is crucial to ensure that ICTs are used as a tool to support care, maintaining close and empathetic communication between professionals, patients, and their families. Additionally, the future evaluation of cost-effectiveness of these interventions in hospital services is essential.

Based on the findings, this study provides guidance for professionals by offering a theoretical framework grounded in scientific evidence for conducting health education with hospitalized patients, empowering them regarding their health issues. For researchers, there is a highlighted need for well- designed randomized clinical trials on the effectiveness of educational interventions conducted in the hospital setting. Furthermore, the evaluation of the cost-effectiveness of these interventions for services is paramount. For managers and policymakers, educational interventions are recommended as effective means to improve patient knowledge and subsequent participation in healthcare. These interventions are also noted for their low or no implementation costs and their potential for patient adherence in healthcare institutions.
